# Unexpected uterine microhabitat of *Schistosoma* infection: a migrant case of female genital schistosomiasis in Europe

**DOI:** 10.1186/s40249-026-01502-0

**Published:** 2026-09-09

**Authors:** Antonio Beltrán-Rosel, Jorge Ligero-López, Lia Ornat-Clemente, Elaine Mejía-Urbáez, Reyes Tébar-Fuentes, Pilar Goñi, Beatriz López-Alonso, Alejandra De Elías-Escribano, Patricio Artigas, María Cecila Fantozzi, María Dolores Bargues

**Affiliations:** 1 Department of Clinical Microbiology, Hospital Clínico Universitario Lozano Blesa, 50009 Saragossa, Spain; 2 Department of Microbiology, Pediatrics, Radiology and Public Health, Faculty of Medicine, University of Zaragoza, 50009 Saragossa, Spain; 3 Group of Water and Environmental Health, Institute of Environmental Sciences (IUCA), 50009 Saragossa, Spain; 4 Department of Clinical Microbiology, Hospital Universitario San Jorge, 22004 Huesca, Spain; 5 Aragón Health Sciences Institute (IACS), 50009 Saragossa, Spain; 6 Department of Gynecology and Obstetrics, Hospital Clínico Universitario Lozano Blesa, 50009 Saragossa, Spain; 7 Department of Surgery, Gynecology and Obstetrics, Faculty of Medicine, University of Zaragoza, 50009 Saragossa, Spain; 8 Department of Pathology, Hospital Clínico Universitario Lozano Blesa, 50009 Saragossa, Spain; 9 Épila Health Center, Épila, 50290 Saragossa, Spain; 10 Department of Parasitology, Faculty of Pharmacy, University of Valencia, Burjassot, 46100 Valencia, Spain; 11 CIBER de Enfermedades Infecciosas, Instituto de Salud Carlos III, 28029 Madrid, Spain; 12 Department of Microbiology, Hospital Universitario San Jorge, Av. de Martínez de Velasco 36, 22004 Huesca, Spain

**Keywords:** *Schistosoma haematobium*, Female genital schistosomiasis, Infertility, Hydrosalpinx, Endometrial biopsy

## Abstract

**Background:**

Female genital schistosomiasis (FGS) is an underrecognized cause of infertility and may remain undiagnosed in non-endemic settings because of nonspecific symptoms and limited sensitivity of routine diagnostic tests.

**Case presentation:**

We report the case of a 30-year-old woman from Côte d’Ivoire who had lived in Mali before migrating to Spain and then presented with primary infertility. Initial fertility workup revealed a large intramural–submucosal leiomyoma. Following myomectomy, histopathological examination unexpectedly identified *Schistosoma* eggs associated with granulomatous inflammation in the myometrial specimen. Initial *Schistosoma* serology and urine microscopy were negative. Hysterosalpingography later demonstrated right hydrosalpinx, prompting reconsideration of schistosomiasis. Repeated urine samples collected after endometrial biopsy revealed *Schistosoma haematobium*-like eggs, confirming the diagnosis. Retrospective review showed previous eosinophilia despite the absence of urinary symptoms or hematuria. The patient was treated with praziquantel, and follow-up samples were negative.

**Conclusions:**

This case highlights FGS as a potentially reversible cause of infertility in migrant women from endemic regions. It also underscores the diagnostic limitations of serology and urine microscopy and the importance of histopathological examination in establishing the diagnosis.

**Graphical Abstract:**

**Figure:**
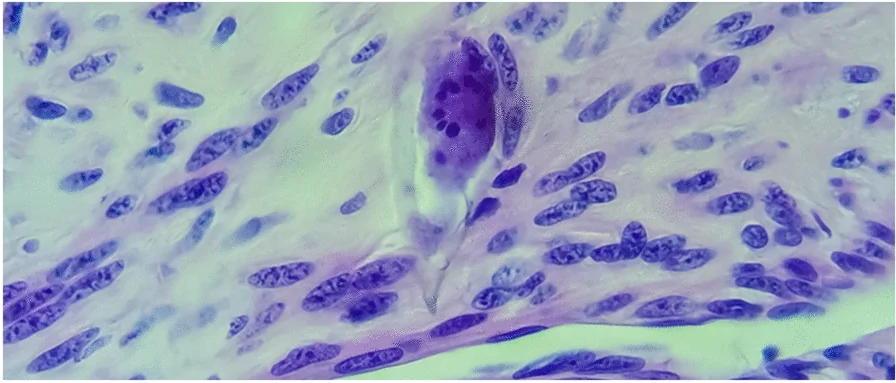

## Background

Infertility is a major global health concern, affecting an estimated 10–15% of couples worldwide and imposing substantial medical, psychological, and socioeconomic burdens. In high-income countries, infertility is commonly associated with delayed childbearing, endocrine disorders, tubal pathology, and failures of assisted reproductive technologies. In contrast, in low- and middle-income countries, infectious diseases remain a leading and often preventable cause [1]. Among these, neglected tropical diseases (NTDs) constitute an underrecognized yet significant contributor to female reproductive morbidity and infertility, particularly in endemic settings [2, 3]. These infections may affect reproductive health through several mechanisms, including chronic inflammation, genital and pelvic tissue damage, tubal pathology, anemia, and other forms of reproductive tract injury, potentially resulting in impaired fertility and adverse pregnancy outcomes [2, 4]. Their impact may be particularly important among women living in resource-limited settings, where the burden of these infections is high and their effects on reproductive health remain insufficiently recognized [4].

Schistosomiasis is one of the most prevalent NTDs worldwide, affecting more than 230 million people, predominantly in sub-Saharan Africa. Urogenital schistosomiasis, caused by *Schistosoma haematobium*, disproportionately affects women of reproductive age and has been increasingly associated with adverse reproductive outcomes, including subfertility and infertility [4, 5]. Female genital schistosomiasis results from the deposition of schistosome eggs in the genital tract, where chronic granulomatous inflammation may cause tissue damage, fibrosis, anatomical distortion, and tubal pathology. Involvement of the upper reproductive tract, including the uterus and fallopian tubes, is particularly difficult to recognize and may remain clinically silent, potentially contributing to infertility without the typical manifestations of genital schistosomiasis [4, 5].

The aim of this report is to describe an unusual case of female genital schistosomiasis (FGS) with uterine involvement in a migrant woman presenting with infertility in a non-endemic setting, and to highlight the diagnostic challenges associated with this atypical presentation.

## Case presentation

### Initial infertility evaluation

We report the case of a 30-year-old woman from Côte d’Ivoire who lived in Yamoussoukro until age 13, then in Bamako, Mali, until 2022, when she moved to Spain. She has since resided continuously in Spain without further travel abroad.

The couple had been attempting to conceive since August 2022 and presented to our infertility unit in early 2025. Her medical, surgical, and family histories were unremarkable, with no toxic habits. One year earlier, she had presented to the emergency department with lower abdominal pain. She reported regular 28-day menstrual cycles, mild dysmenorrhea, and no dyspareunia; gynecological examination was normal.

Initial fertility workup revealed normal ovarian reserve parameters, including anti-Müllerian hormone (AMH) levels of 3.07 ng/ml (normal range: 0.07–7.35), thyroid-stimulating hormone (TSH) of 1.5 mU/L (normal range: 0.27–4.2), and prolactin of 6.78 ng/ml (normal range: 4.79–23.3). Transvaginal ultrasound demonstrated a 72 × 72 mm type 3 anterior intramural–submucosal leiomyoma, in contact with but not distorting the endometrial cavity and located away from both tubal ostia, with limited peripheral vascularization. Both ovaries had normal morphology, with five antral follicles each.

The partner’s initial semen analysis was within normal limits; however, a subsequent evaluation showed mild reductions in sperm concentration and morphological quality, with a preserved total motile sperm count.

### Incidental histological finding and initial evaluation

**Fig. 1:**
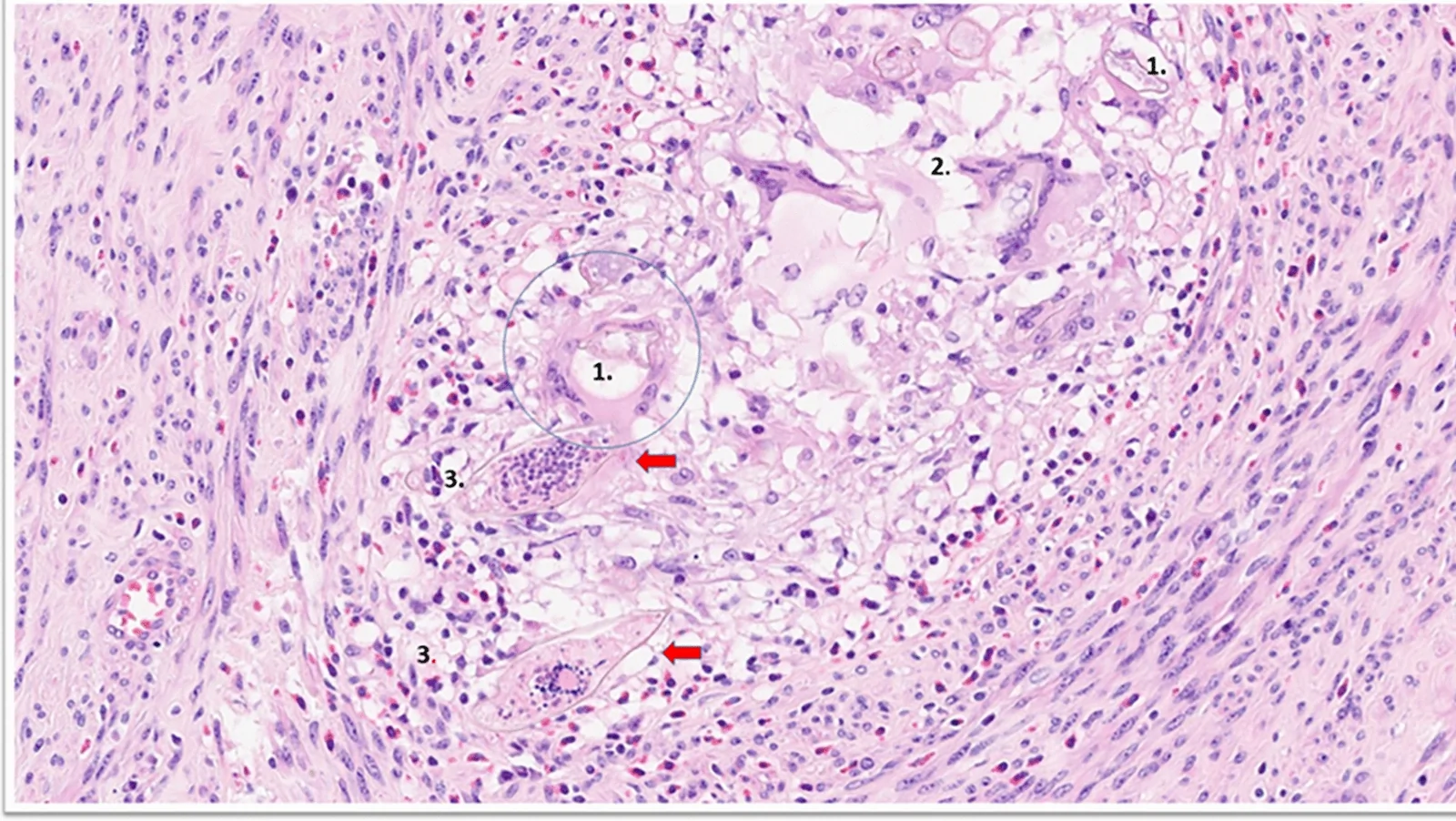
Hematoxylin and eosin-stained histological section of a resected uterine leiomyoma at 20× magnification. 1. Multinucleated giant cells of foreign body-type containing phagocytosed eggs. 2. Granulomatous reaction. 3. Chronic inflammatory infiltrate with eosinophils, plasma cells, and lymphocytes around the *Schistosoma* eggs

In April 2025, the patient underwent an open myomectomy. Intraoperative findings confirmed a large anterior intramural fibroid (approximately 9 cm) and a smaller posterior subserosal fibroid. The endometrial cavity was not entered during the procedure. Histopathological examination revealed intersecting fascicles of uniform spindle cells arranged in smooth muscle bundles, consistent with an intramural leiomyoma. Scattered, rounded structures suggestive of *Schistosoma* eggs were identified sparsely and diffusely throughout the myometrium, accompanied by a mild chronic inflammatory infiltrate of lymphocytes, eosinophils, and plasma cells and multinucleated foreign body-type giant cells, consistent with a granulomatous reaction. Many eggs showed dystrophic calcification. **(**Fig. 1**).** The pathologist notified the treating clinician of these findings.

Given this unusual finding, a consultation with the infectious diseases service was requested. In June 2025, an extended laboratory workup was performed. Biochemical parameters were within normal limits. However, the complete blood count showed mild abnormalities, including low mean corpuscular volume (MCV, 80.9 fL; reference range 82–98 fL), low mean corpuscular hemoglobin (MCH, 25.6 pg; 27–33 pg), elevated red cell distribution width (RDW, 15.4%; 11.5–14.5%), relative neutropenia (36.7%; 40–75%), relative lymphocytosis (46%; 20–45%), and mild eosinophilia (0.5 × 10⁹/L [9.1%]; reference range 0–0.5 × 10⁹/L; 0–6%).

Serological testing for human immunodeficiency virus (HIV), human T-cell lymphotropic virus (HTLV), hepatitis B and C, *Strongyloides*, *Entamoeba histolytica*, and *Schistosoma* antibodies (ELI.H.A *Schistosoma*, ELITechGroup, Signes, France) was performed. All results were negative, except for seropositivity for hepatitis B surface and core antibodies (anti-HBs and anti-HBc). Parasitological examination of a single urine sample (collected in the morning after mild physical activity) was negative. Microbiological evaluation of vaginal and endocervical swabs demonstrated normal flora, with the presence of *Lactobacillus* species. Polymerase chain reaction (PCR) assays for *Chlamydia trachomatis*, *Neisseria gonorrhoeae*, *Trichomonas vaginalis*, and *Mycoplasma genitalium* (Alinity m STI assay, Abbott Molecular, Des Plaines, IL, USA) were all negative. In the absence of peripheral absolute eosinophilia, negative serology for *Schistosoma*, and no parasite detection in urine, genitourinary schistosomiasis was considered unlikely.

### Diagnosis and treatment of schistosomiasis

**Fig. 2:**
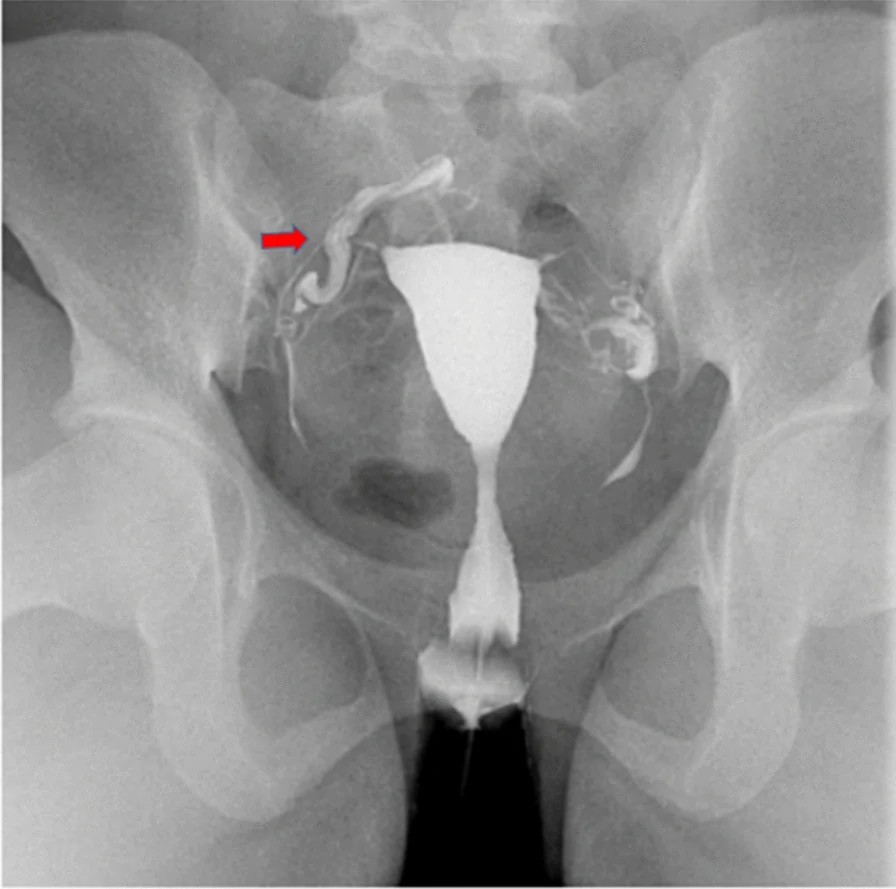
Hysterosalpingography showing the uterine cavity with normal morphology. The right fallopian tube shows segmental dilatation along its course (arrow) but remains patent. The left fallopian tube demonstrates normal morphology and is patent

In November 2025, hysterosalpingography revealed a midline anteflexed uterus with a normal cervical canal and endometrial cavity. The right fallopian tube was patent but showed hydrosalpinx, whereas the left tube was patent with normal morphology **(**Fig. 2**).**

Given these findings, and in the absence of any other identifiable cause of infertility, schistosomiasis was reconsidered as a potential diagnosis. As the biopsy specimen obtained in April had been fixed in formalin and was unsuitable for molecular analysis, a new endometrial biopsy was performed during a subsequent gynecological consultation in January 2026.

**Fig. 3:**
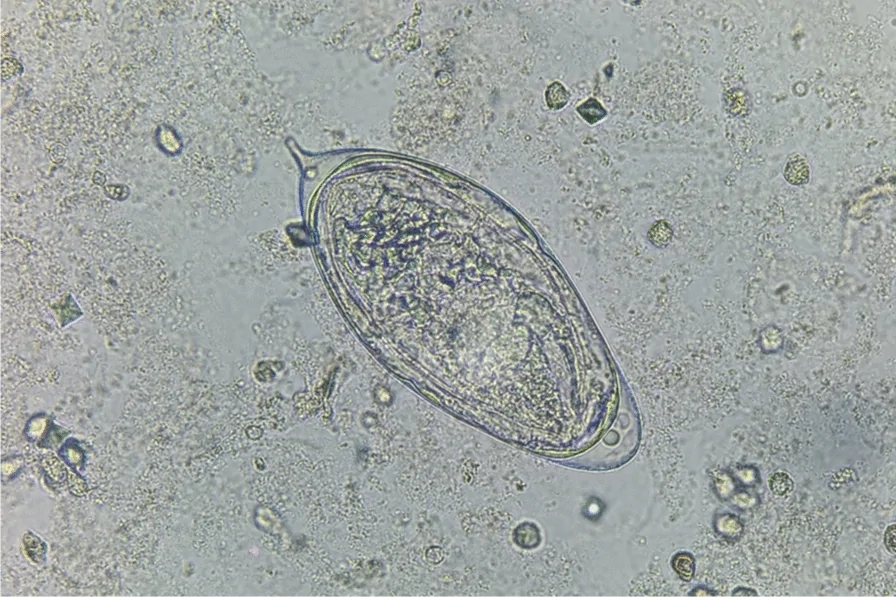
Centrifuged urine sample showing a *Schistosoma* egg with a characteristic terminal spine

In the days following the procedure, three consecutive urine (collected, again, in the morning after mild physical activity) and stool samples were collected. All stool samples tested negative. Urinalysis revealed turbidity and varying degrees of hematuria, and microscopic examination of the sediment identified several *S. haematobium*-like eggs **(**Fig. 3**).** A review of previous laboratory records from August 2023 showed marked eosinophilia, with absolute and relative eosinophil counts of 1.3 × 10⁹/L and 20.6%, respectively.

Following these findings, the patient was re-interviewed. She reported dysmenorrhea as the most prominent past symptom, along with occasional, poorly defined abdominal discomfort, but denied any history of dysuria or hematuria. On January 23, 2026, she was treated with a single dose of praziquantel (40 mg/kg).

### Further characterization and follow-up

**Fig. 4:**
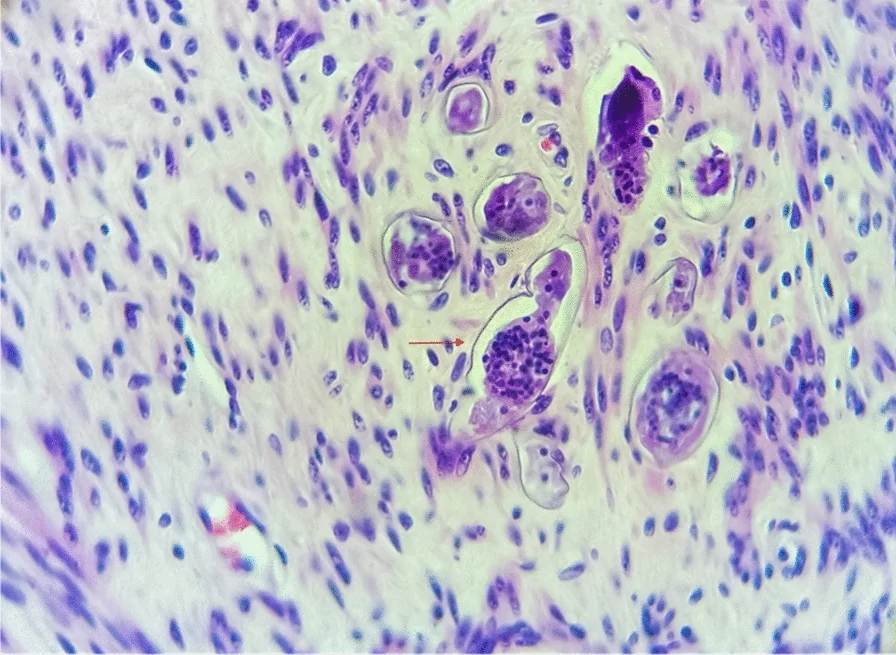
Hematoxylin and eosin-stained histological section of a resected uterine leiomyoma. In some instances, the egg can be observed in its full length. Note its morphology, characterized by a broader central region and tapering at both ends (arrow)

Once the diagnosis of schistosomiasis was established, the histological sections from the leiomyoma excised in April 2025, along with additional sections of the surgical specimen, were re-examined. Measurements were obtained from miracidia-containing eggs. Although complete eggs were infrequently visualized in histological sections, maximum width was measured in all identifiable eggs, and length was recorded when the egg was visualized in full longitudinal section. The eggs showed a mean width of 44.84 µm (range: 37.5–65 µm) and a mean length of 109.58 µm (range: 70.0–137.5 µm) **(**Fig. 4**).** To assess urinary tract involvement and potential hepatic complications, hepatic ultrasound (February 24, 2026), cystoscopy (March 26, 2026), and urological ultrasound (April 10, 2026) were performed, all of which were unremarkable.

At this stage, schistosomiasis was investigated in her partner, who was also originally from Mali. A complete blood count, three urine samples, and three stool samples for parasitological examination, as well as serological testing for *Schistosoma* antibodies (ELI.H.A *Schistosoma*, ELITechGroup, Signes, France), were requested. The complete blood count was within normal limits, and all urine and stool samples were negative; however, *Schistosoma* serology was positive. A second set of three urine and three stool samples was subsequently obtained, again yielding negative results. In the absence of parasitological evidence, active *Schistosoma* infection was considered unlikely, and no treatment was administered.

The endometrial biopsy fixed in 70% ethanol and two of the three post-biopsy urine samples were sent to the Parasitology Department of the University of Valencia for molecular and morphometric studies. PCR amplification and sequencing using mitochondrial and ribosomal markers are ongoing to investigate *S. haematobium* and potential hybrid forms in the urine and endometrial samples. These findings will be reported separately.

As part of follow-up, both an endometrial biopsy (March 18, 2026) and a urine sample (March 26, 2026) were negative for *Schistosoma* eggs. At present, no structural causes of infertility have been identified. A repeat hysterosalpingography performed in June 2026 showed persistent right hydrosalpinx with tubal obstruction. The couple is currently on the waiting list for in vitro fertilization.

## Discussion

Infertility is a major global public health issue, affecting millions of individuals worldwide. A systematic review and meta-analysis estimated the cumulative lifetime prevalence of infertility at 17.5% [6], although estimates vary across regions and populations. In sub-Saharan Africa, a meta-analysis including 16 studies and 22,303 participants reported a pooled prevalence of 16.98% [1]. Despite comprehensive diagnostic evaluation, the etiology of infertility remains unexplained in approximately 10–30% of couples [7].

Leiomyomas are considered the sole identifiable cause of infertility in only 1–3% of cases [8]. However, submucosal or intracavitary fibroids have been associated with impaired reproductive outcomes [9], while most women with fibroids remain fertile. In the present case, the intramural–submucosal leiomyoma was unlikely to be the primary cause of infertility. Its size, anterior location, lack of endometrial cavity distortion, limited vascularization, and preserved tubal patency make a significant impact on fertility improbable. Notably, histopathological analysis of the leiomyoma specimen, performed as part of the infertility workup, unexpectedly revealed the likely underlying cause of infertility: *Schistosoma* infection.

Schistosomiasis is frequently identified as an incidental finding in biopsies performed for unrelated indications. When genital involvement occurs, it predominantly affects the cervix and, to a lesser extent, the fallopian tubes [10, 11]. Female genital schistosomiasis (FGS) results from egg deposition in the cervix, uterus, fallopian tubes, and adjacent pelvic tissues, leading to chronic granulomatous inflammation, fibrosis, and anatomical distortion. Proposed mechanisms of infertility include tubal obstruction, impaired sperm migration, altered endometrial receptivity, microvascular damage, and immune-mediated tissue injury [12].

Epidemiological data support a clear association between *S. haematobium* endemicity and increased infertility rates. Up to 75% of infected individuals may develop lesions involving both the urinary and genital tracts [13]. In endemic settings, schistosomiasis-related tubal disease has been reported in approximately 40% of women with infertility in Zanzibar (Pemba Island, Tanzania), while in Niger, 38.5% of infertile women had urinary infection with *S. haematobium*, which was associated with a higher prevalence of genital lesions compared with uninfected individuals [14]. Despite this, its contribution to the global burden of infertility remains underrecognized.

Importantly, many individuals with genital schistosomiasis do not excrete *S. haematobium* eggs in urine [15]; therefore, negative urine microscopy does not exclude the diagnosis. The most commonly reported symptoms include vaginal discharge, itching, leucorrhea, contact bleeding, menstrual irregularities, dyspareunia, and, in some cases, severe pelvic pain [16]. However, none of these manifestations were observed in our patient. Although endometrial biopsy demonstrated parasitic involvement, the presence of hydrosalpinx on hysterosalpingography suggests possible tubal involvement, which cannot be excluded.

The diagnosis of FGS remains challenging due to the absence of a reference standard. It relies primarily on colposcopy or histological examination, which, although specific, require trained personnel and are associated with procedural limitations. Even in endemic settings, many healthcare professionals are unable to recognize typical lesions [16]. Lesions affecting the vagina and cervix are more readily identified, whereas involvement of the upper genital tract—including the uterus, fallopian tubes, and ovaries—is considerably more difficult to detect [12], as illustrated in our case. Importantly, these diagnostic challenges often result in delayed recognition and may lead to irreversible reproductive sequelae [17].

In endemic regions, FGS frequently coexists with sexually transmitted infections (STIs), contributing to chronic cervicovaginal and pelvic inflammation and further compromising reproductive health [18, 19]. These overlapping conditions may synergistically increase the risk of infertility. In a study conducted in Madagascar, 35% of women with *S. haematobium* infection had coexisting STIs—such as *Neisseria gonorrhoeae*, *Chlamydia trachomatis*, *Mycoplasma genitalium*, or *Trichomonas vaginalis*—compared with 17% of men, with stronger association observed at higher parasite burdens [18]. Furthermore, a meta-analysis reported that individuals with schistosomiasis have a 2.3-fold increased risk of acquiring HIV compared with uninfected individuals [20]. In our patient, serological and molecular testing excluded these infections, thereby supporting *Schistosoma* infection as the most likely cause of infertility.

Eosinophilia is present in only a minority of patients with chronic schistosomiasis (approximately 25%) and is often absent in long-standing infections or in those with a low parasite burden, indicating that this parameter alone is insufficient for diagnosis or screening [21]. HTLV co-infection may suppress eosinophil responses and facilitate uncontrolled proliferation of *Strongyloides stercoralis* [22]. In fact, serial blood analyses showed a progressive decline in both absolute and relative eosinophil counts from 2023 onwards. For this reason, HTLV serology was requested and was negative. The observed decrease in eosinophils is therefore more likely related to the natural course of schistosomiasis, in which reduced antigenic stimulation over time may lead to diminished eosinophilic activity.

In diagnostic studies, the sensitivity for detecting *S. haematobium* infection varies widely, ranging from approximately 21% to 71% depending on the assay used (e.g., enzyme-linked immunosorbent assay (ELISA), indirect fluorescent antibody test (IFAT)) [23]. In migrants and travelers with confirmed infection, the sensitivity of serological testing for immunoglobulin G (IgG) antibodies against *S. haematobium* has been reported to be as low as 44% [24]. In our case, serological testing for *Schistosoma* was negative, which may reflect both the chronicity of infection and the intrinsic limitations of the assay. Importantly, a negative serological result does not exclude *S. haematobium* infection.

The diagnosis of parasitic infections in tissue samples can be challenging. Pathologists may have limited exposure to parasitic morphology, and recognition of characteristic features can be difficult, particularly in atypical locations. At the same time, parasitologists are not routinely trained to interpret histological sections. Furthermore, parasites in tissue often appear fragmented or sectioned obliquely, with morphological features that differ from those observed in conventional samples such as urine or stool. Although the increasing use of molecular and antigen-based assays has reduced reliance on microscopy, traditional morphological evaluation remains essential, particularly when molecular techniques are unavailable or unsuitable, as in formalin-fixed specimens [25].

In our case, *Schistosoma* eggs were initially identified in the myometrial specimen by the pathologist, whereas confirmation proved more challenging for the parasitology team. Additional histological sections were required to establish the diagnosis. Although earlier treatment with praziquantel could have been considered once structures suggestive of *Schistosoma* eggs had been identified in a patient originating from an endemic area, the initial histopathological findings were regarded as inconclusive because of the unusual uterine location and the difficulty in confirming the morphology after multidisciplinary review. The negative serology, the absence of absolute eosinophilia, and the negative urine microscopy further reduced the clinical suspicion of active schistosomiasis. Consequently, additional investigations were undertaken before treatment was initiated. Once *Schistosoma* eggs were demonstrated in urine, praziquantel was administered without delay.

This case highlights the critical importance of multidisciplinary collaboration in achieving accurate diagnosis and appropriate management. It may seem unexpected that the initial urine sample tested negative for parasites, whereas three subsequent samples collected after the endometrial biopsy were positive for *Schistosoma* eggs. Notably, the patient had not previously reported chronic abdominal pain or hematuria despite these findings. One possible explanation is that the endometrial biopsy, together with urine collection in the days following the procedure, may have facilitated the release or mobilization of eggs from the genital tract into the urinary system, thereby increasing their detectability. However, this hypothesis remains speculative. Post-treatment follow-up included the analysis of three additional urine samples, a complete blood count, and repeat serological testing for *Schistosoma*. Nevertheless, the optimal strategy for assessing treatment response has not been clearly established.

Clinical management should be interpreted in the context of a non-endemic setting, where schistosomiasis is uncommon and clinical suspicion may be lower, particularly when presentations are atypical. In our setting, infections most frequently involve the urinary tract, which may have further contributed to the initial diagnostic approach in this case. In the June 2025 blood test, the absolute eosinophil count was at the upper limit of the reference range, whereas the relative eosinophil count was elevated (9%). This finding was not initially considered suggestive of parasitic infection, as current Spanish recommendations place greater diagnostic value on absolute eosinophilia than on isolated relative eosinophilia in travelers and migrants with suspected imported parasitic diseases [26]. In retrospect, previously documented episodes of absolute eosinophilia were not taken into account during the initial evaluation, and earlier recognition of these findings might have led to a more timely diagnosis and treatment.

Likewise, only a single urine sample was initially examined for parasitological analysis, and no stool samples were requested. This likely reflected the low initial clinical suspicion of schistosomiasis, given the negative serology, the absence of absolute eosinophilia, and the negative urine microscopy. Although examination of three consecutive urine samples is recommended to increase the diagnostic sensitivity for urinary schistosomiasis, this was not performed at the initial assessment. In retrospect, collection of three urine samples together with stool examination would have represented a more appropriate diagnostic approach and might have facilitated an earlier diagnosis.

The management of the patient's partner also deserves consideration. Although he was asymptomatic, had no eosinophilia, and repeated parasitological examinations (two sets of three urine and three stool samples) remained negative, anti-*Schistosoma* serology was positive, indicating previous exposure. At the time, these findings were interpreted as not supporting active schistosomiasis, and treatment was therefore deferred after multidisciplinary discussion. However, current expert recommendations support treatment of previously untreated individuals with positive *Schistosoma* serology, even in the absence of parasitological confirmation. In retrospect, empirical praziquantel treatment could also have been considered in this case.

## Conclusions

In endemic areas, schistosomiasis remains an important and often underrecognized contributor to infertility and should be considered in the diagnostic workup. This is particularly relevant in non-endemic settings, where a lower index of suspicion and limited clinical experience may delay diagnosis. While lesions of the vagina and cervix are more readily identified, involvement of the upper genital tract remains considerably more difficult to detect; notably, our case, likely acquired in Mali, highlights uterine involvement and underscores the diagnostic challenges in such presentations.

## Data Availability

No datasets were generated or analysed during the current study.

## Abbreviations

AMH: Anti-Müllerian hormone
ELISA: Enzyme-linked immunosorbent assay
FGS: Female genital schistosomiasis
HIV: Human immunodeficiency virus
HTLV: Human T-cell lymphotropic virus
IFAT: Indirect fluorescent antibody test
IgG: Immunoglobulin G
MCH: Mean corpuscular hemoglobin
MCV: Mean corpuscular volume
NTDs: Neglected tropical diseases
PCR: Polymerase chain reaction
RDW: Red cell distribution width
STIs: Sexually transmitted infections
TSH: Thyroid-stimulating hormone
